# Circular nucleic acids at the host-virus interface: from immune modulation to therapeutic innovation

**DOI:** 10.3389/fimmu.2026.1751068

**Published:** 2026-01-29

**Authors:** Jiacheng Lou, Jing Liu, Danlu Zhang, Yuchao Hao

**Affiliations:** 1 Department of Pediatrics, the Second Hospital of Dalian Medical University, Dalian, China; 2 Department of Hematology, the Second Hospital of Dalian Medical University, Dalian, China; 3 Human Resources Department, the Second Hospital of Dalian Medical University, Dalian, China

**Keywords:** antiviral response, circular RNA (circRNA), covalently closed circular DNA (cccDNA), hepatitis B virus (HBV), hepatitis delta virus (HDV), innate immunity, viral infection, viral pathogenesis

## Abstract

Nucleic acids, long regarded as linear polymers, are now recognized to also exist in circular forms with profound biological significance in both eukaryotic hosts and viruses. This review synthesizes emerging insights into the diverse roles of circular RNAs (circRNAs) and other circular nucleic acids in viral infection and immunity. We first discuss the biogenesis and functions of host-derived circRNAs, emphasizing their complex interplay with innate immunity. These molecules display a striking duality—capable of activating antiviral defenses through pattern recognition receptors such as RIG-I and PKR, yet also exploited by viruses as modulators of immune evasion. We then examine how viral evolution has repeatedly converged on circular architectures, from the minimalist circular RNA genomes of viroids and hepatitis D virus (HDV) to transiently or covalently circularized RNA and DNA viral genomes. A particular focus is placed on hepatitis B virus (HBV), whose covalently closed circular DNA (cccDNA) serves as the persistent nuclear template driving viral replication and chronic infection. We summarize current understanding of cccDNA transcriptional regulation, host factors influencing its activity, and clinical biomarkers such as serum HBV RNA and HBcrAg that reflect cccDNA dynamics. Finally, we highlight the biotechnological applications of circularity, including circRNA-based vaccines offering superior stability and durable antigen expression, and circular nucleic acid probes for viral diagnostics. Collectively, this review positions circular nucleic acids as central players at the host–virus interface, shaping immunity, persistence, and the next generation of antiviral strategies.

## 1 Introduction

The study of virology is fundamentally an exploration of the interactions between viral and host genomes (1). For decades, this field has been dominated by a linear perspective, focusing on the linear DNA and RNA molecules that constitute the genetic material of most known organisms and their viral pathogens. However, accumulating evidence has revealed that circularity is not a biological curiosity but a widespread and functionally critical structural motif for nucleic acids across all domains of life. In eukaryotes, a vast and diverse class of endogenous non-coding RNAs, known as circular RNAs (circRNAs), are generated from the back-splicing of pre-mRNAs (2). These covalently closed loops were initially dismissed as splicing artifacts but are now recognized as stable and abundant molecules with crucial roles in gene regulation (3).

Concurrently, the viral world offers a parallel narrative of circularity. Many viruses have evolved to possess or generate circular nucleic acid genomes or replication intermediates. These range from the small, pathogenic circular RNAs of plant viroids and the human Hepatitis Delta Virus (HDV), to the large circular DNA genomes of various DNA viruses, and even the non-covalent cyclization of linear RNA genomes in viruses like flaviviruses and HIV-1 (4–6). This convergent evolution underscores the inherent advantages conferred by a circular topology, such as resistance to exonuclease degradation, facilitation of rolling-circle replication, and precise regulation of gene expression.

The intersection of these two worlds—endogenous host circRNAs and the circular nucleic acids of viruses—creates a complex and dynamic battleground that defines the outcome of infection. Host circRNAs are deeply entwined with the innate immune system, where they can act as both sentinels that trigger antiviral alarms and as regulators that fine-tune these responses (7, 8). Viruses, in turn, have developed strategies to mimic, exploit, or subvert these host pathways. Perhaps the most compelling example of a pathogen harnessing the power of circularity for its survival is the Hepatitis B Virus (HBV). The persistence of HBV, which chronically infects hundreds of millions of people worldwide, is primarily sustained by the formation of a stable, nuclear, plasmid-like covalently closed circular DNA (cccDNA) episome that serves as the transcriptional template for viral replication. In addition, HBV DNA can integrate into the host genome, where it remains replication-defective but nevertheless contributes to long-term viral persistence and pathogenesis (9, 10).

This review provides a detailed discourse on the intricate interplay between circular nucleic acids and viral infections. We will explore the biogenesis of host circRNAs and their dualistic role in antiviral immunity. We will then survey the diverse strategies employed by viruses that utilize circular genomes or intermediates. A substantial focus will be placed on the molecular biology of the HBV cccDNA, including its regulation, its role in pathogenesis, and the development of clinical biomarkers to monitor its activity. Finally, we will discuss how the inherent stability and functional properties of circular nucleic acids are inspiring a new generation of vaccines and diagnostics, fundamentally changing our approach to combating viral diseases.

## 2 The biogenesis and functional repertoire of endogenous circular RNAs

Before delving into their role in virology, it is essential to understand the nature of endogenous host circRNAs. Unlike their linear mRNA counterparts, which are generated by canonical splicing of pre-mRNAs, circRNAs are produced through a non-canonical process known as “back-splicing.” In this reaction, a downstream 5’ splice site is covalently linked to an upstream 3’ splice site, forming a continuous, closed-loop structure that lacks the 5’ cap and 3’ poly(A) tail characteristic of linear mRNAs (11). The covalently closed circular structure of circRNAs confers exceptional resistance to exonuclease-mediated degradation while simultaneously shielding their RNA ends from both RNases and end-recognizing pattern recognition receptors such as retinoic acid-inducible gene I (RIG-I) (3).

The process of back-splicing is not a random error but is often a regulated event, facilitated by specific sequence elements and protein factors. One key mechanism involves the pairing of inverted repeat elements, such as Alu sequences, located in the introns flanking the circularizing exons. This base-pairing brings the relevant splice sites into close proximity, favoring the back-splicing reaction. Furthermore, specific RNA-binding proteins have been identified as crucial regulators of circRNA biogenesis. For instance, the double-stranded RNA-binding proteins NF90 and NF110 have been demonstrated to promote the formation of circRNAs by binding to these flanking intronic inverted Alu repeats. By stabilizing the RNA duplex structure, NF90/NF110 effectively act as molecular clamps, enhancing the efficiency of the back-splicing machinery to generate specific circRNA isoforms (12). This regulated production underscores that circRNAs are intended functional products of gene expression.

**Figure 1:**
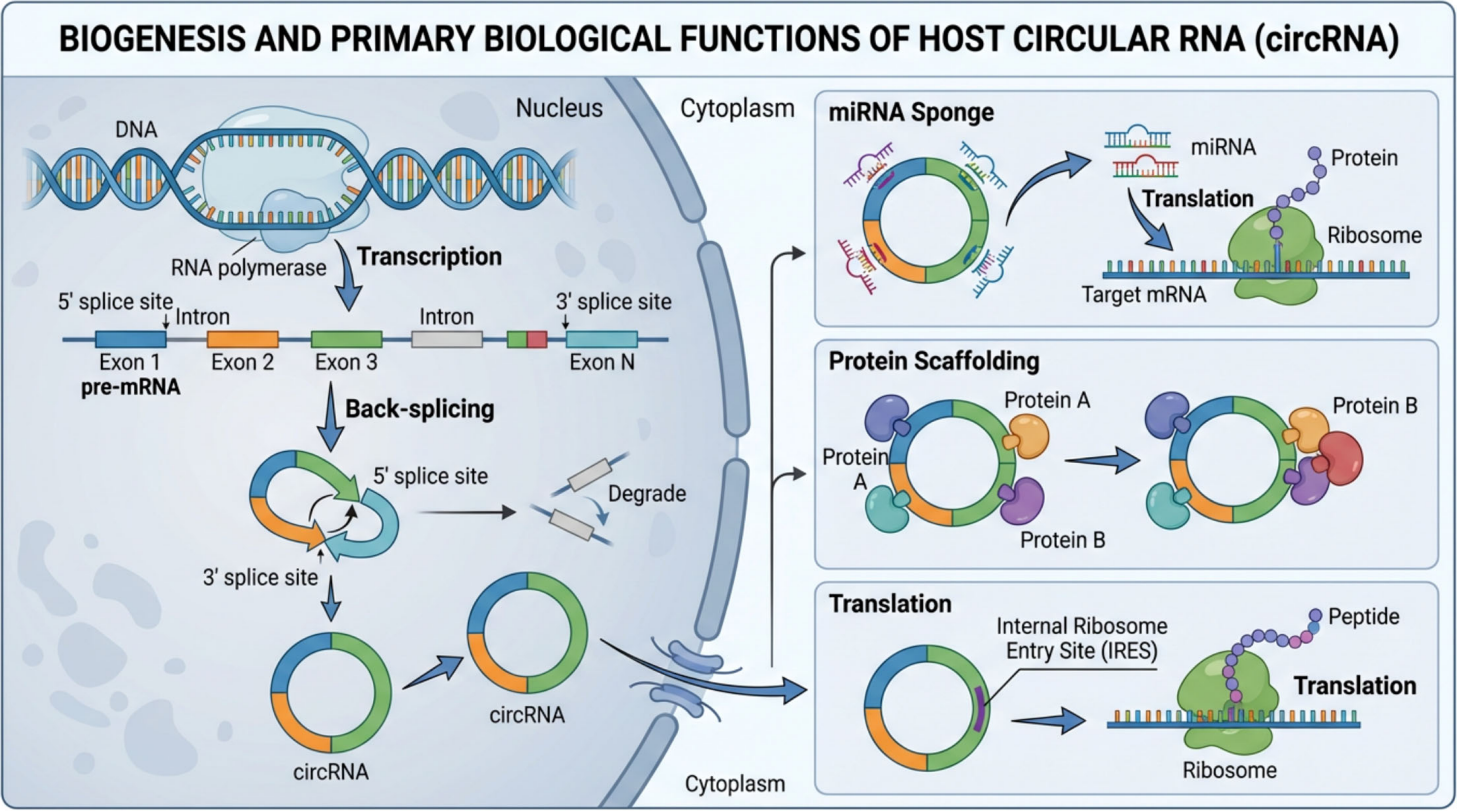
Biogenesis and primary biological functions of host circular RNAs (circRNAs). CircRNAs are generated from precursor mRNAs through a back-splicing reaction in the nucleus, in which a downstream 5′ splice site is covalently joined to an upstream 3′ splice site, producing a closed circular RNA molecule. Linear RNA byproducts are typically degraded, whereas circRNAs are exported to the cytoplasm due to their enhanced stability. In the cytoplasm, circRNAs exert diverse biological functions. They can act as microRNA (miRNA) sponges, sequestering miRNAs and thereby modulating the translation of target mRNAs. CircRNAs also function as protein scaffolds, facilitating or stabilizing interactions between distinct proteins. In addition, a subset of circRNAs can serve as templates for cap-independent translation via internal ribosome entry sites (IRESs), leading to the production of functional peptides. Together, these mechanisms highlight the multifaceted regulatory roles of circRNAs in gene expression and cellular homeostasis.

Once produced, circRNAs can exert their biological functions through a variety of mechanisms (**Figure 1**). They can act as “sponges” for microRNAs (miRNAs) or RNA-binding proteins, sequestering these molecules and thereby regulating their activity on other targets (13). They can also serve as protein scaffolds, bringing together multiple proteins to facilitate complex formation or enzymatic reactions. In some cases, circRNAs containing an internal ribosome entry site (IRES) can be translated into proteins, adding another layer to their functional complexity (14). A compelling example of translational regulation, though observed in the context of a viral RNA, illustrates a potential mechanism for host circRNAs. The circularized genome of flaviviruses has been shown to inhibit the translation of host mRNAs, suggesting that circular topology itself can influence ribosomal activity, potentially by competing for translation initiation factors or by physically impeding scanning ribosomes (6). The diverse functions of these stable, covalently closed molecules position them as significant players in cellular homeostasis and, consequently, in the cellular response to pathological insults like viral infection.

## 3 The dichotomous role of circular RNAs in antiviral innate immunity

**Figure 2:**
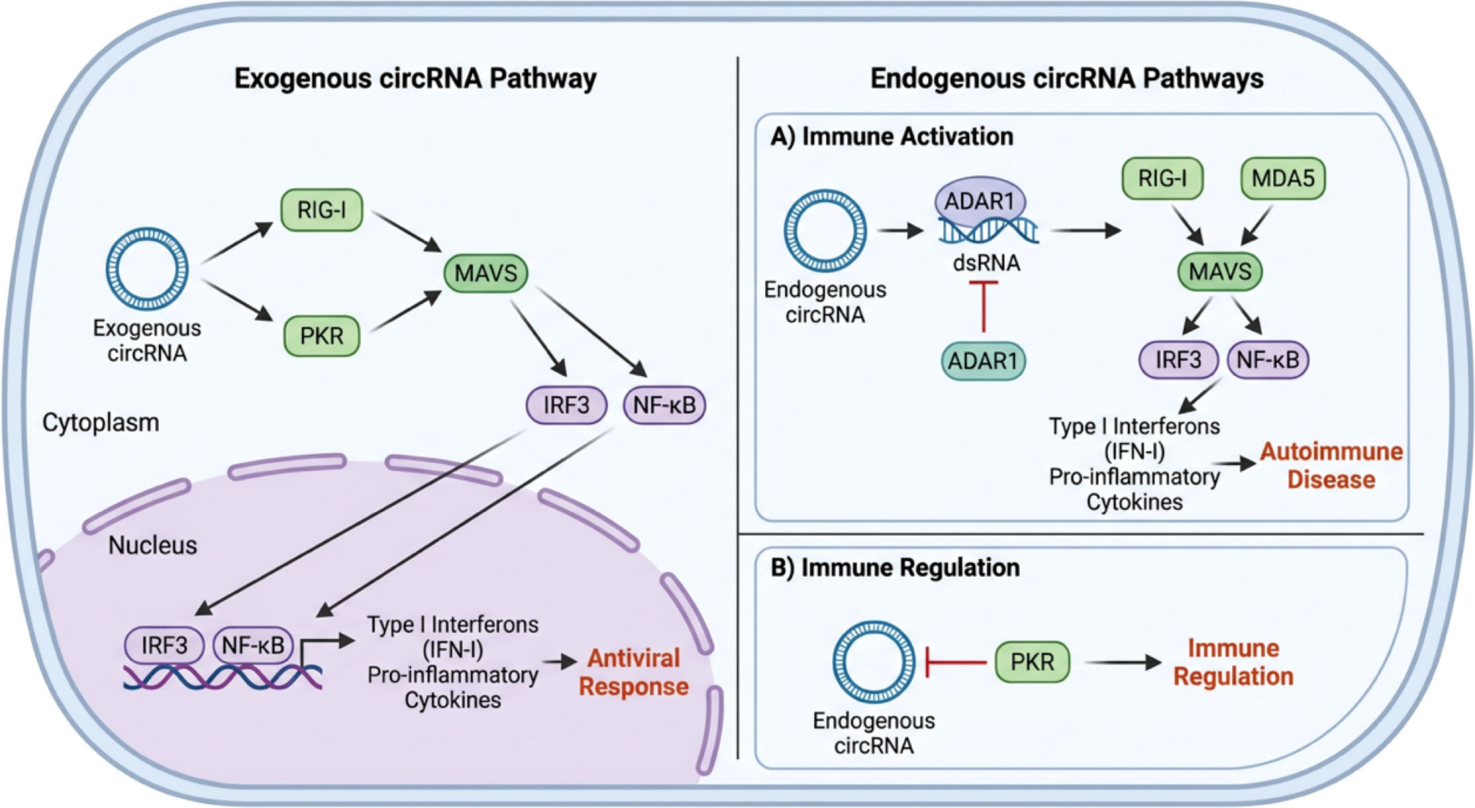
Distinct innate immune sensing and regulatory pathways of exogenous and endogenous circular RNAs (circRNAs). Exogenous circRNAs introduced into the cytoplasm are recognized as non-self nucleic acids and sensed by pattern recognition receptors, including RIG-I and PKR, leading to MAVS-dependent activation of downstream signaling pathways. This results in phosphorylation and nuclear translocation of IRF3 and activation of NF-κB, thereby inducing type I interferons and pro-inflammatory cytokines and promoting an antiviral immune response (left panel). In contrast, endogenous circRNAs generated by host back-splicing are subject to stringent self-tolerance mechanisms (right panels). **(A)** Under certain conditions, such as impaired RNA editing, endogenous circRNAs may form double-stranded RNA structures and activate innate immune sensors, triggering type I interferon responses and pro-inflammatory cytokine production, potentially contributing to autoimmune pathology. **(B)** Alternatively, endogenous circRNAs can exert immune-regulatory functions by interacting with sensors such as PKR, thereby dampening excessive innate immune activation and maintaining immune homeostasis.

The innate immune system constitutes the first line of defense against invading pathogens, relying on a diverse array of pattern recognition receptors (PRRs) to detect non-self molecular patterns such as viral nucleic acids and to trigger antiviral immune responses (15). Traditionally, innate immune sensing has been viewed through the paradigm of distinguishing “self” from “non-self” (**Figure 2**). However, accumulating evidence suggests a broader concept in which PRRs also recognize “altered self”—host nucleic acids that acquire abnormal structural or chemical features. For example, hyperediting of self-dsRNA by ADAR1 generates multiple I-U mismatches that act to prevent Melanoma Differentiation-Associated protein 5 (MDA5) oligomerization (16). In the absence of ADAR1 editing, long dsRNA stem loops can form that activate MDA5, leading to aberrant interferon responses (17, 18). Similarly, improperly processed or RNase-digested self-RNAs that expose 5′-triphosphate termini can be recognized by RIG-I, triggering type I interferon signaling (19). This shift from the “non-self” to the “altered self” model underscores that innate immune sensors monitor both exogenous and dysregulated endogenous nucleic acids, thereby maintaining a delicate balance between antiviral defense and self-tolerance.

PRRs act as essential sensors of the immune system. They play a pivotal role in bridging innate and adaptive immune responses by initiating host defense mechanisms against invading pathogens while maintaining immune homeostasis (20). Upon recognition of pathogen-associated molecular patterns (PAMPs) or damage-associated molecular patterns (DAMPs), PRRs trigger a series of downstream signaling cascades. Through these coordinated signaling pathways, PRRs orchestrate the early innate immune response and shape subsequent adaptive immunity, thereby ensuring both rapid pathogen clearance and immune regulation. PRRs are host proteins encoded in the germline, existing either as membrane-bound or cytosolic entities. PRRs encompass several major families, including Toll-like receptors (TLRs), RIG-I–like receptors (RLRs), C-type lectin receptors (CLRs), nucleotide-binding oligomerization domain (NOD)–like receptors (NLRs), and others. Canonically, mammalian PRRs are thought to be specific for a single PAMP—for example, TLR4 binds LPS and TLR5 binds flagellin, RIG-I binds double-stranded RNA (15).

**Figure 3:**
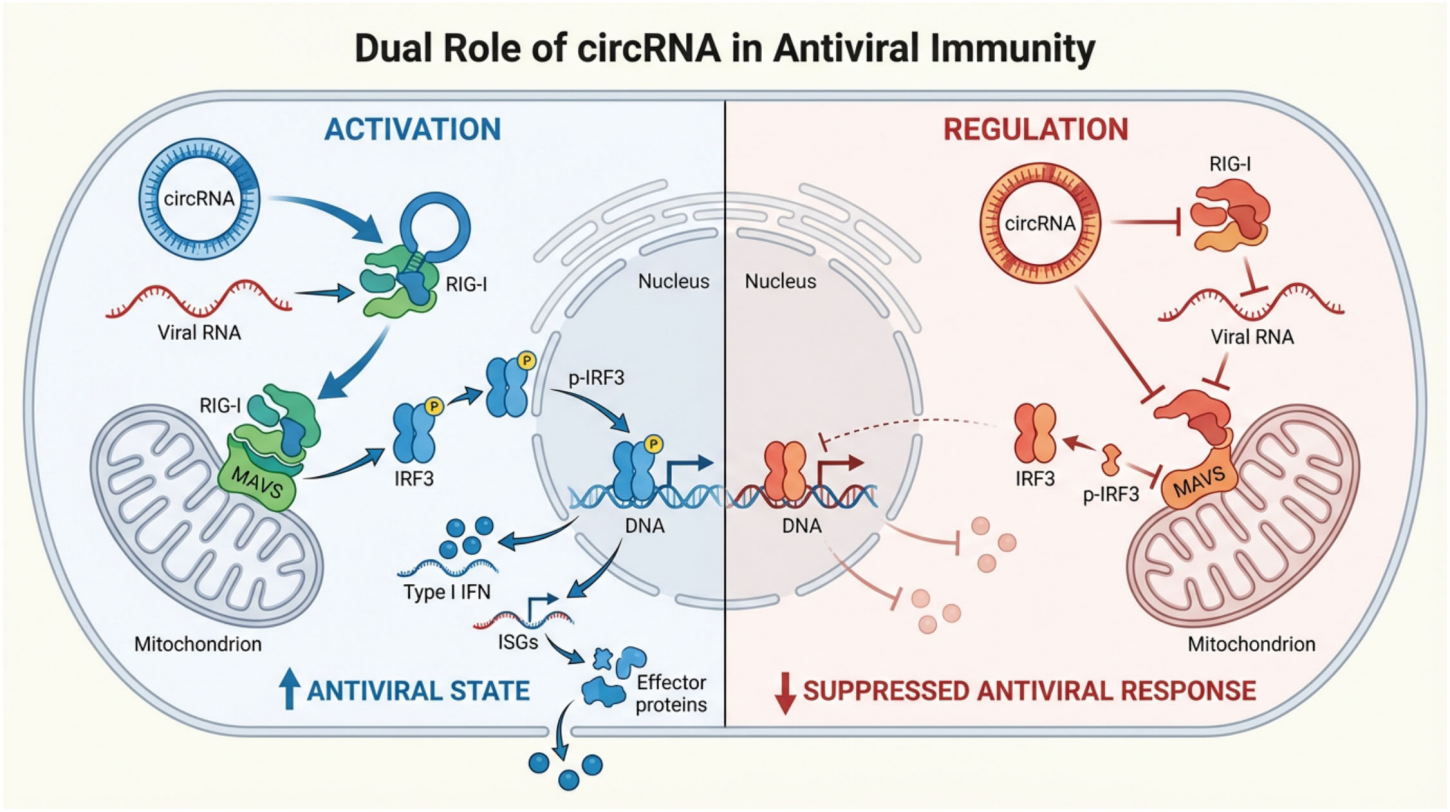
Dual roles of circular RNAs (circRNAs) in antiviral innate immunity. CircRNAs exert context-dependent functions in antiviral immunity by either promoting or restraining innate immune signaling. Left panel (Activation): Exogenous or aberrant circRNAs, together with viral RNAs, can be recognized by the cytosolic pattern recognition receptor RIG-I, leading to activation of the MAVS signaling complex at the mitochondria. This triggers phosphorylation of IRF3, its nuclear translocation, and subsequent induction of type I interferons (IFNs) and interferon-stimulated genes (ISGs), thereby establishing an antiviral state. Right panel (Regulation): In contrast, endogenous circRNAs can negatively regulate antiviral responses by interacting with RIG-I or downstream signaling components, limiting IRF3 activation and interferon production. This regulatory function contributes to the suppression of excessive innate immune activation and maintenance of immune homeostasis. Together, these opposing activities highlight circRNAs as dynamic modulators of antiviral immunity rather than passive byproducts of RNA processing.

Given their unique structure and potential to form double-stranded regions, circRNAs are intrinsically positioned to interact with this system (**Table 1**). This interaction is profoundly dichotomous (**Figure 3**): circRNAs can act as potent triggers of antiviral immunity, but they can also serve as sophisticated modulators, a duality that is central to the host-virus conflict (3).

**Table 1:** Comparative immune-sensing properties of different classes of circular RNAs.

Category	Representative molecules	Nucleic acid type	Primary localization	Major PRRs involved	Innate immune activation	Biological/Immunological implications	Key references
Host-derived circular RNAs	Endogenous circRNAs	RNA	Cytoplasm/Nucleus	Typically none (immune-tolerant)	Low to absent under homeostasis	Maintain immune self-tolerance; function in post-transcriptional regulation	(21)
Aberrant or synthetic circular RNAs	*In vitro*–generated circRNAs with impurities	RNA	Cytoplasm	RIG-I, MDA5, TLR7/8	Moderate to high (context-dependent)	Tunable immunogenicity for vaccines and immunotherapies	(21, 22)
Viral circular RNAs	HDV RNA	RNA	Cytoplasm/Nucleus	Predominantly MDA5	Strong type I/III IFN induction	Restricts viral spread but fails to eradicate established infection	(23)
cccDNA (covalently closed circular DNA)	HBV cccDNA	DNA	Nucleus	Generally evades cGAS–STING sensing	Minimal direct innate immune activation	Sustained antigen expression; immune tolerance and chronic persistence	(24, 25)
Circular DNA (viral or synthetic episomes)	Episomal vectors	DNA	Nucleus	Conditional cGAS activation	Context-dependent	Applications in gene therapy with immune risk considerations	(26)

### 3.1 Circular RNAs as inducers of antiviral signaling

The cellular machinery for detecting foreign nucleic acids is highly sensitive to features that deviate from typical host molecules. While the host produces its own circRNAs, exogenous circRNAs, such as those that might be delivered by a virus or a synthetic vaccine, can be potent immunostimulants. Studies have shown that cells have evolved a splicing-dependent mechanism for the discrimination of endogenous and exogenous circRNA, using RIG-I as a cytoplasmic sensor of exogenous circRNA (21). While circRNA does not contain the triphosphate motif canonically required for RIG-I activation, it has been suggested that upon binding to its ligand, RIG-I undergoes a conformational change and initiates a signaling cascade that culminates in the production of type I interferons (IFNs) and other pro-inflammatory cytokines, establishing a robust antiviral state in the host cell and its neighbors (21). This finding forms the basis for the development of circRNA-based vaccine adjuvants and platforms.

Another critical PRR involved in sensing circRNAs is Protein Kinase R (PKR). PKR is activated by binding to double-stranded RNA (dsRNA), a common hallmark of viral replication. Many endogenous circRNAs, particularly those formed from adjacent exons, can contain imperfect intramolecular double-stranded regions due to inverted repeat sequences or local folding. These structures can be sufficient to activate PKR. Furthermore, the degradation of circRNAs, although slow, can produce linear dsRNA fragments that are also potent PKR activators (27). Once activated, PKR phosphorylates the eukaryotic initiation factor 2 alpha (eIF2α), leading to a global shutdown of protein synthesis, which serves to halt the production of both host and viral proteins, thereby restricting viral spread. The activation of PKR by endogenous circRNAs has been implicated not only in antiviral defense but also in pathological conditions like the autoimmune disease Systemic Lupus Erythematosus (SLE), where an overabundance of circRNA-PKR complexes may contribute to chronic inflammation (28).

To prevent aberrant activation of innate immunity by its own circRNAs, the cell has evolved safeguard mechanisms. One such critical mechanism is the chemical modification of RNA. N6-methyladenosine (m^6^A) is the most abundant internal modification on eukaryotic mRNA and has also been found on circRNAs. It has been demonstrated that m^6^A modification of endogenous circRNAs helps the immune system to distinguish “self” from “non-self.” The presence of m^6^A marks these molecules as host-derived, preventing them from being recognized and targeted by PRRs like RIG-I. A lack of m^6^A modification on circRNAs leads to their recognition as foreign, triggering a potent type I IFN response. This highlights a sophisticated system of RNA epigenetics that is crucial for maintaining immune tolerance to self-circRNAs while remaining vigilant against foreign ones (29).

Beyond m^6^A modification, 2′-O-methylation has emerged as another critical RNA mark for self–nonself discrimination. Host mRNAs typically contain both N^7^-methylation and 2′-O-methylation at their 5′ cap structures, catalyzed by nuclear and cytoplasmic methyltransferases, respectively. In contrast, many viral RNAs lack 2′-O-methylation, rendering them more susceptible to recognition by RIG-I or MDA5 and to translational suppression mediated by interferon-induced proteins with tetratricopeptide repeats (IFITs). Notably, mutants of West Nile virus, poxvirus, and coronavirus deficient in 2′-O-methyltransferase activity exhibit marked attenuation in interferon-competent cells but retain pathogenicity in the absence of type I interferon signaling. These findings demonstrate that 2′-O-methylation not only distinguishes host RNA from foreign RNA but also functions as a viral strategy to evade IFIT-mediated antiviral restriction (30, 31).

Moreover, the compartmentalization and encapsulation of viral genomes and replication intermediates represent additional strategies for immune evasion, as many viruses sequester their nucleic acids within membrane-bound replication organelles or viral capsids, thereby shielding them from cytosolic PRRs (32, 33).

### 3.2 Circular RNAs as modulators and targets of viral subversion

Beyond acting as direct triggers, circRNAs also function as regulators that can dampen or fine-tune the immune response. This modulatory role often involves their ability to bind and sequester key proteins in the immune signaling pathway. The interaction with PKR is a prime example of this regulatory capacity. While some circRNAs activate PKR, others can bind to it without inducing its full kinase activity. By acting as a competitive inhibitor or a “sink” for PKR, these circRNAs can prevent its activation by bona fide viral dsRNA, thereby modulating the intensity of the antiviral response. This creates a delicate balance where circRNAs can set the threshold for PKR activation, preventing an overzealous immune reaction that could be detrimental to the host (28).

Viruses, being master manipulators of host cell machinery, have evolved mechanisms to exploit this regulatory layer of circRNA biology for their own benefit. A clear example is provided by the Epstein-Barr virus (EBV), a human herpesvirus. EBV produces its own circRNA, known as circBART2.2, within infected cells. This viral circRNA has been shown to facilitate immune evasion by binding to and stabilizing the mRNA of PD-L1, a critical immune checkpoint protein. The resulting upregulation of PD-L1 on the surface of infected cells allows them to inhibit the function of cytotoxic T cells, effectively shielding the virus from immune clearance (34). This demonstrates a sophisticated strategy where a virus-encoded circular RNA co-opts host pathways to create an immunosuppressive microenvironment.

Another intriguing example comes from the Human Immunodeficiency Virus 1 (HIV-1). The HIV-1 accessory protein Vpr has been found to induce the formation of a specific class of host circRNAs known as circular intronic transcripts (ciTRANs). The induction of these specific circRNAs by a viral protein suggests that they may play a role in the viral life cycle, possibly by modulating host gene expression or immune responses in a manner that favors viral replication and persistence (35). Similarly, the host factor NLRP12, an innate immune sensor, produces a truncated protein isoform (NLRP12-119aa) that has been found to inhibit rhabdovirus replication, indicating that host-derived factors can possess direct antiviral functions, potentially modulated by or interacting with circRNA networks (36). These examples illustrate that the interplay between host circRNAs and viruses is a complex dance of activation, regulation, and subversion, with the outcome of infection often hinging on the balance of these interactions.

### 3.3 Circularity as a convergent strategy in the viral kingdom

The adoption of a circular nucleic acid architecture is not unique to the host but is a recurring theme throughout the viral world, manifesting in diverse forms across a wide range of viruses that infect bacteria, plants, and animals. This convergent evolution highlights the powerful selective advantages offered by circularity, including enhanced stability, unique replication mechanisms, and novel ways to regulate viral gene expression and interact with the host.

## 4 Viruses with circular RNA genomes and intermediates

The simplest and perhaps most ancient examples of pathogenic circular molecules are viroids. These are small, non-encapsidated, single-stranded circular RNA molecules that cause a variety of diseases in plants. Lacking any protein-coding capacity, viroids exert their pathogenicity solely through the structure and sequence of their RNA genome, which allows them to hijack host cellular machinery for replication and interfere with host gene regulation, often by acting as templates for the host’s RNA silencing machinery (4). A key feature of many viroids is their ability to self-cleave and re-ligate via embedded ribozyme domains, a property that is essential for their rolling-circle mode of replication (37). The discovery of viroid-like circular RNAs in fungi has further expanded the known host range of these minimalist pathogens, suggesting they are a more widespread class of infectious agents than previously thought (38).

In humans, the Hepatitis Delta Virus (HDV) stands out as a unique viroid-like pathogen. HDV possesses a small, single-stranded, circular RNA genome and requires the surface antigen of HBV for its packaging and transmission, making it a satellite virus. Despite its minimal genome, HDV infection can lead to severe and rapidly progressive liver disease. The circular HDV RNA is a potent inducer of innate immune responses, leading to robust activation of type I and type III interferons (IFN-β and IFN-λ), which restrict cell division–mediated spread of HDV genomes. However, these immune responses have limited impact on viral RNA replication within already infected, quiescent hepatocytes. Consistently, therapeutic administration of IFN-α or IFN-λ alone can transiently suppress HDV replication but rarely achieves sustained viral clearance, with a high proportion of patients developing severe or fulminant hepatitis (39). HDV can also establish latent infections that may be reactivated upon subsequent HBV infection, underscoring the persistent nature of its circular genome (40). Moreover, HDV replication depends on multiple host factors, including CAD, JAK1, and ADAR, which have been identified as potential therapeutic targets (41–43).

Even viruses with conventionally linear RNA genomes can utilize circularity. Flaviviruses, a family that includes Dengue, Zika, and Yellow Fever viruses, possess a single-stranded, positive-sense RNA genome that is linear within the virion. However, upon entering the host cell, the genome circularizes through long-range RNA-RNA interactions between conserved sequences at the 5’ and 3’ ends. This non-covalent cyclization is crucial for initiating translation and for positioning the viral RNA-dependent RNA polymerase to begin replication. This transient, functional circularization also appears to suppress the translation of host mRNAs, giving the virus a competitive advantage (6). Similarly, the genomic RNA of HIV-1 has also been observed to form circularized structures, which may play a role in regulating reverse transcription or viral gene expression (5).

Circular DNA is a hallmark of many viruses, from bacteriophages to complex animal viruses. Bacteriophages, the viruses that infect bacteria, exhibit incredible diversity in their genetic material, with many possessing circular single-stranded or double-stranded DNA genomes (44). The replication of these genomes often involves specific regulatory elements, as seen in the M13 phage, where the regulation of RNA transcription is tightly controlled to manage the viral life cycle (45).

In the plant kingdom, Geminiviruses are a major family of pathogens characterized by their circular single-stranded DNA genomes. Upon infection, these viruses replicate in the host nucleus, producing double-stranded DNA intermediates that serve as templates for transcription. The host plant defends itself by recognizing these viral DNA and RNA molecules and initiating a potent RNA silencing (RNA interference) response to degrade viral transcripts and control the infection (46).

A novel form of antiviral defense involving circular DNA has been discovered in insects. In response to RNA virus infection, insect cells can use viral RNA as a template to generate small, circular viral DNA (cvDNA) molecules through a reverse transcriptase-dependent mechanism. These cvDNAs then serve as templates for the production of siRNAs that target the viral RNA for degradation, establishing a durable, DNA-based antiviral RNAi memory (47). This represents a fascinating intersection of reverse transcription, circular DNA formation, and RNA interference in invertebrate immunity. Finally, retroviruses like the Feline Leukemia Virus, while having an RNA genome, replicate through a DNA intermediate that can exist in circular forms within the host cell nucleus prior to integration into the host chromosome (48). The existence of these circular forms is a critical step in the retroviral life cycle. This wide-ranging adoption of circular nucleic acids across disparate viral families underscores its fundamental utility in replication, persistence, and host interaction.

Among all human viruses that utilize a circular genome, the HBV presents one of the greatest challenges to global public health. The chronicity of HBV infection, which can lead to cirrhosis and hepatocellular carcinoma (HCC), is inexorably linked to the establishment and long-term stability of a unique nuclear structure: the cccDNA. This molecule, a viral minichromosome, is the master template for all viral gene expression and the ultimate source of viral persistence, making it the central target for any curative therapy (9, 49).

## 5 The central role of covalently closed circular DNA

**Figure 4:**
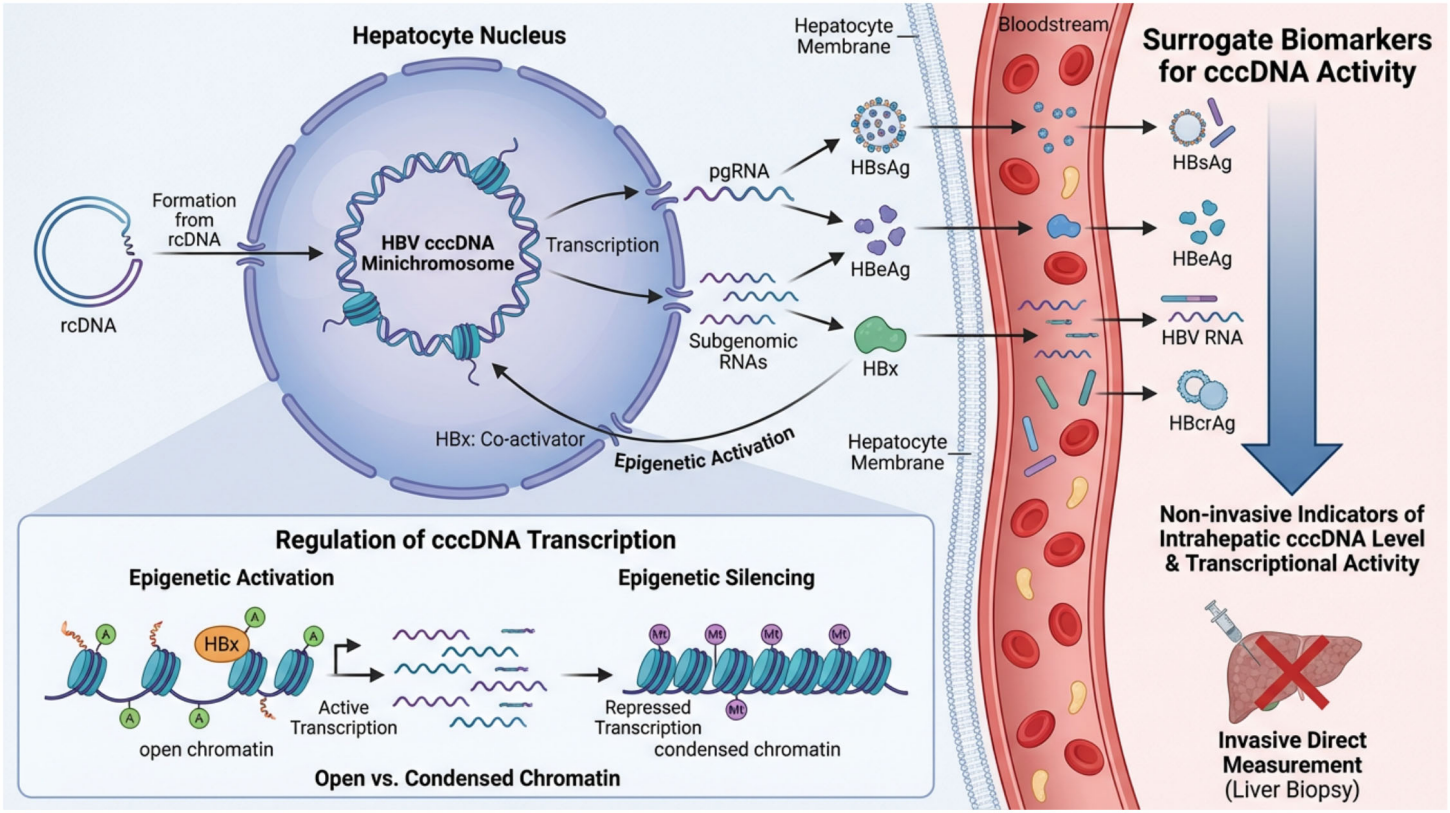
The key points of HBV cccDNA. Following entry into hepatocytes, relaxed circular DNA (rcDNA) is transported to the nucleus and converted into covalently closed circular DNA (cccDNA), which is organized into a chromatinized minichromosome. This cccDNA minichromosome serves as the transcriptional template for all viral RNAs, including pregenomic RNA (pgRNA) and subgenomic RNAs encoding viral proteins such as hepatitis B surface antigen (HBsAg), hepatitis B e antigen (HBeAg), core antigen (HBcAg), and HBx. HBx functions as a key co-activator that promotes epigenetic activation of cccDNA by maintaining an open chromatin state. The transcriptional activity of cccDNA is dynamically regulated by epigenetic modifications, with histone acetylation favoring active transcription and chromatin condensation leading to transcriptional silencing. Viral RNAs and proteins are subsequently exported from hepatocytes into the bloodstream. Because direct quantification of intrahepatic cccDNA requires invasive liver biopsy, circulating viral products—including HBsAg, HBeAg, HBV RNA, and HBcrAg—are widely used as non-invasive surrogate biomarkers reflecting cccDNA abundance and transcriptional activity.

The cccDNA is a class of circular DNA molecules where both strands are topologically closed without any free 5’ or 3’ ends. Unlike linear DNA or gap-containing circular DNA, cccDNA is chemically continuous, with both complementary strands linked end-to-end via phosphodiester bonds to form a highly stable closed-loop structure. The most well-known biological background of cccDNA comes from HBV infection (**Figure 4**). Upon infecting a hepatocyte, the relaxed circular DNA genome of the HBV virion is transported to the nucleus. There, host DNA repair enzymes convert it into a supercoiled, protein-free cccDNA molecule.

The stability of the cccDNA pool is the primary barrier to curing chronic HBV. While nucleotide/nucleoside analogues (NAs), the current standard of care, can effectively suppress viral replication and reduce viral loads, they do not act on the cccDNA itself. Consequently, upon cessation of therapy, the cccDNA pool can readily reactivate transcription, leading to a rebound of viremia (50). Therefore, achieving a functional cure (sustained HBsAg loss) or a sterilizing cure (complete eradication of all viral forms) for HBV is critically dependent on either silencing the transcriptional activity of cccDNA or eliminating the cccDNA pool altogether (51, 52).

### 5.1 The complex regulation of cccDNA transcription and activity

The transcriptional activity of the cccDNA minichromosome is not constitutive but is subject to a complex web of regulation by both host and viral factors, creating a battleground within the infected hepatocyte nucleus. Numerous host factors have been identified that can suppress cccDNA transcription. For example, the protein arginine methyltransferase 5 (PRMT5) (53), mitochondrial sirtuin SIRT3 (54), and the long non-coding RNA HOXA-AS2 (55). Conversely, some host factors can be co-opted by the virus to enhance its transcription. The High Mobility Group A1 (HMGA1) protein, a chromatin architectural factor, has been shown to positively regulate HBV transcription, and its expression is upregulated by the viral HBx protein, creating a positive feedback loop that favors viral gene expression (56).

This complex regulation leads to significant heterogeneity in viral activity. Many hepatocytes may harbor cccDNA, but only a fraction of these episomes are transcriptionally active at any given time, suggesting that local chromatin environments and stochastic fluctuations in regulatory factors play a critical role (57, 58). This heterogeneity poses a significant challenge for therapies aimed at silencing cccDNA, as dormant episomes may escape treatment and reactivate later.

### 5.2 Clinical monitoring of cccDNA activity: the role of viral biomarkers

Directly measuring the amount and activity of cccDNA in the liver requires an invasive liver biopsy, which is not feasible for routine patient monitoring. Therefore, the field has focused intensely on developing reliable, non-invasive serum biomarkers that can serve as surrogates for intrahepatic viral activity. One of the most promising biomarkers is serum HBV RNA. These RNA molecules are primarily encapsidated pgRNA that are secreted from infected cells in virion-like particles. Because they are transcribed directly from the cccDNA, their levels in the blood are thought to closely reflect the transcriptional activity of the cccDNA pool in the liver (59–61). Circulating HBV RNA has shown significant clinical utility, with studies demonstrating its ability to predict HBeAg seroconversion, the risk of developing resistance to lamivudine, and the likelihood of achieving HBsAg clearance after treatment (62–64).

### 5.3 The persistence puzzle: cccDNA dynamics and integrated HBV DNA

Although highly stable, the intrahepatic cccDNA pool is not static and can be diluted through hepatocyte proliferation, representing a key non-cytolytic mechanism of viral clearance during immune recovery or effective therapy (65, 66). Emerging evidence suggests that cccDNA undergoes dynamic turnover, with concurrent loss and replenishment sustaining chronic infection (67).

HBV persistence is further reinforced by viral DNA integration into the host genome. Although replication-defective, integrated HBV DNA can act as a stable template for HBsAg transcription, such that elimination of cccDNA alone may not achieve functional cure (68). This mechanism explains persistent HBsAg positivity despite undetectable viremia during long-term nucleos(t)ide analogue therapy, a hallmark of occult HBV infection associated with increased hepatocellular carcinoma risk (69–71).

### 5.4 cccDNAs as immunomodulatory entities

cccDNA exemplifies how circular nucleic acids can act as persistent immunomodulatory entities at the host–pathogen interface. Unlike linear viral genomes, HBV cccDNA exists as a chromatinized, episomal minichromosome that is highly resistant to degradation and largely insulated from canonical innate immune surveillance pathways, enabling sustained viral antigen transcription in the absence of active replication (72, 73). This circular topology, together with nuclear compartmentalization, allows cccDNA to evade cytosolic DNA sensing mechanisms such as cGAS–STING, thereby minimizing innate immune activation while maintaining chronic antigen exposure—a defining feature of immune tolerance and T cell dysfunction in persistent HBV infection (74).

In a broader immunological context, cccDNA aligns with an emerging paradigm in which circular nucleic acids—both viral and endogenous—exert distinct immune effects through enhanced stability, altered sensing, and controlled transcriptional output. Analogous to endogenous circRNAs, which generally escape RIG-I–like receptor activation unless aberrantly processed, cccDNA demonstrates how circular nucleic acid topology can decouple durability from immunogenicity. Conceptualizing cccDNA as an active immune-modulatory platform rather than a passive genetic reservoir provides a unifying framework that links HBV persistence to the expanding field of circular RNA–based vaccines and therapeutics, where balancing stability, antigen expression, and innate immune engagement is critical for effective immune intervention (75).

## 6 Therapeutic and diagnostic innovations inspired by circular nucleic acids

The growing appreciation for the unique physicochemical and biological properties of circular nucleic acids has catalyzed the development of innovative biotechnological applications, particularly in the fields of vaccinology, diagnostics, and gene therapy. The inherent stability and distinct immunological features of circular molecules are being harnessed to overcome the limitations of traditional linear platforms.

### 6.1 Circular RNA as a next-generation vaccine platform

The recent success of linear mRNA vaccines has revolutionized the field, but challenges related to stability and delivery remain (76). CircRNA vaccines have emerged as a highly promising next-generation platform that addresses some of these limitations. By engineering a gene of interest into a circular RNA molecule, a vaccine can be created that is highly resistant to exonuclease degradation, leading to a much longer intracellular half-life compared to its linear counterpart (22, 77). This prolonged stability results in more durable and sustained expression of the target antigen, which can elicit a stronger and more long-lasting immune response from a smaller dose (78).

This concept has been successfully translated into practice. A circular RNA vaccine encoding the monkeypox virus surface antigen has been shown to elicit potent and protective neutralizing antibody responses, demonstrating its efficacy against this emerging pathogen (79). Similarly, a circRNA-based vaccine has proven effective in protecting against Zika virus (ZIKV) infection in preclinical models (80). The intrinsic adjuvant properties of circRNAs, which can stimulate PRRs like RIG-I, can be fine-tuned during the design process to optimize the balance between antigen expression and immunostimulation, making circRNA a versatile and powerful platform for developing vaccines against a wide range of infectious diseases.

### 6.2 Novel diagnostic and therapeutic strategies

The unique topology of circular molecules also lends itself to the creation of novel diagnostic tools. For example, circular DNA probes have been designed for the rapid and highly sensitive detection of specific RNA viruses (81). These probes can hybridize to their target viral RNA, and upon successful binding, can be ligated and amplified using rolling circle amplification, a technique that generates a massive signal from a single binding event. This allows for the detection of very low levels of viral RNA without the need for reverse transcription, offering a potential advantage in speed and simplicity for point-of-care diagnostics (82).

Furthermore, the principles of circularity are being integrated into therapeutic strategies. The technology known as CPER (Circular Permutation-based virus-like particle Engineering) allows for the construction of recombinant RNA viruses by manipulating their genomes in a circular permutation format, which can facilitate research and the development of viral vectors for gene therapy or oncolytic applications (83). In the context of HBV, the ultimate circular target—the cccDNA—is the focus of intense therapeutic development. Novel strategies aim to directly eliminate or permanently silence this episome. These include the use of engineered nucleases, delivered via mRNA, that are designed to specifically recognize and cut the cccDNA, leading to its degradation (84). Other approaches utilize RNA interference (RNAi) technologies, such as siRNAs (e.g., ARC-520), to degrade all viral transcripts, including the pgRNA, thereby cutting off the supply chain for both virion production and cccDNA replenishment (85). Combining these direct-acting agents with immunotherapies represents the most promising path toward a functional cure for chronic hepatitis B (52, 86).

## 7 Strategies for circular RNA production and their biological implications

The unique structure confers stability to circRNA, making it an attractive platform for RNA therapeutics, vaccines, and sustained protein expression. Over the past decade, multiple *in vitro* and cellular strategies have been developed to efficiently generate circRNAs, each with distinct advantages and limitations in terms of circularization efficiency, scalability, product purity, and compatibility with translation mechanisms.

### 7.1 *In vitro* transcription coupled with self-splicing ribozymes

Self-splicing ribozyme-based approaches represent one of the earliest and most widely adopted strategies for circRNA production. Among these, the permuted intron–exon (PIE) system that developed by Wesselhoeft et al. in 2018, derived from group I or group II introns, enables co-transcriptional circularization of target RNA sequences through sequential transesterification reactions (22). The utilization of self-splicing ribozymes in RNA circularization offers several advantages including enhanced circularization efficiency of linear RNA precursors, simplified reaction conditions, and purification methods.

Despite these advantages, ribozyme-based strategies suffer from intrinsic limitations. Residual intronic or ribozyme-derived sequences are often retained in the final circRNA product, potentially introducing immunostimulatory motifs or interfering with RNA folding and translation (87). Moreover, circularization efficiency typically declines with increasing RNA length, limiting the scalability of this approach for large therapeutic circRNAs (88). These drawbacks have driven efforts to minimize ribozyme scars or develop alternative circularization strategies with greater structural precision.

### 7.2 Enzymatic ligation and splint-assisted circularization

A number of methods for enzymatic ligation of synthetic oligonucleotides have been developed. Enzymatic ligation approaches rely on RNA or DNA ligases, such as T4 RNA ligase 1 (T4 Rnl 1), T4 RNA ligase 2 (T4 Rnl 2), or T4 DNA ligase (T4 Dnl), to covalently join the 5′ and 3′ termini of linear RNA transcripts, all encoded in the genome of bacteriophage T4 (89, 90). To enhance ligation efficiency, complementary DNA or RNA splints are frequently employed to spatially juxtapose RNA ends, thereby facilitating intramolecular ligation.

Enzymatic ligation offers superior control over junction sequence fidelity and typically yields circRNAs with minimal extraneous sequences, resulting in higher product purity. However, under this approach, the 5′ terminus requires phosphorylation to provide the functionality necessary for ligation. In the majority of publications, protocols for T4 DNA ligase as well as RNA ligase-mediated circularization of short RNAs (<500 nt) are described. Large substrates in kb scales require different strategies, due to structural challenges. Therefore, as mentioned above, for circularization of large RNAs, protocols using the PIE strategy may be the better alternative (90). Moreover, the enzymatic ligation efficiency is highly dependent on RNA secondary structure and splint design, necessitating careful optimization.

### 7.3 Chemical circularization strategies

Chemical ligation methods connect RNA termini through non-enzymatic reactions, providing theoretical flexibility in chemical modification and sequence design. Those in most cases can be chemically synthesized ensuring homogenous 5′- and 3′-ends. The chemical circularization methods rely on short precursor RNA sequences, synthesized via phosphoramidite chemistry (91). In consequence, the so-far chemically synthesized circRNAs encoded relatively short sequences and have limited use in the context of therapeutic gene delivery (92). Moreover, these approaches often exhibit limited efficiency and are prone to side reactions, leading to heterogeneous products. And the introduction of non-native linkages may compromise RNA stability or translational capacity and could unpredictably affect immune recognition (93). As a result, chemical circularization currently plays a limited role in circRNA production for immunological or therapeutic applications.

### 7.4 Cellular expression and post-transcriptional circularization systems

An alternative strategy for circRNA generation involves engineered cellular expression systems that exploit host-mediated back-splicing or self-splicing mechanisms (94). By incorporating circularization elements into expression vectors, circRNAs can be produced intracellularly without *in vitro* ligation (95). These systems more closely resemble endogenous circRNA biogenesis and may confer favorable biological compatibility. Nevertheless, circularization efficiency in cellular systems is highly dependent on host-specific splicing machinery and sequence context, leading to variability in yield and product uniformity (96). Additionally, precise control over RNA modifications, purity, and translational output remains challenging, limiting current use primarily to exploratory or experimental settings (77).

### 7.5 Impact of production strategies on immunogenicity and translational performance

The immunological behavior of circRNAs is strongly influenced by their production methods. While circRNAs are generally less immunostimulatory than linear RNAs, residual linear RNA species, aberrant junctions, or extraneous sequences can activate innate immune sensors such as RIG-I and Toll-like receptors (97). Production strategies that minimize such features, particularly enzymatic ligation-based approaches, are therefore preferred for immunological applications.

CircRNAs support cap-independent translation through internal ribosome entry sites, m^6^A-mediated initiation, or rolling-circle translation mechanisms. The efficiency of antigen expression is closely linked to junction integrity and RNA structure, both of which are shaped by the chosen circularization strategy. These parameters are critical for vaccine efficacy, where durable antigen presentation and controlled innate immune activation determine the magnitude and quality of adaptive immune responses.

### 7.6 Implications of circular nucleic acids for adaptive immune regulation

Circular nucleic acids can influence adaptive immune responses primarily through sustained antigen availability and indirect modulation of antigen-presenting cell (APC) function. Owing to their enhanced stability and prolonged intracellular persistence, circular RNAs can support durable antigen expression, thereby promoting prolonged MHC class I and II presentation and shaping the magnitude and quality of CD8^+^ and CD4^+^ T-cell responses (22). This property has been experimentally exploited in circRNA-based vaccine platforms, where extended antigen production enhances T-cell priming and memory formation compared with linear mRNA counterparts (98).

In chronic viral infections, circular nucleic acids such as HBV cccDNA or HDV RNA contribute to continuous low-level antigen expression, which may drive T-cell dysfunction and exhaustion rather than effective clearance (23). Persistent antigen presentation in the absence of robust innate co-stimulation favors tolerogenic or exhausted T-cell states, a hallmark of chronic HBV and HDV infection. These observations underscore that circular nucleic acids can shape adaptive immunity in a context-dependent manner, promoting either protective immunity or immune tolerance depending on antigen load, duration, and accompanying innate immune signals.

## 8 Conclusion

The biological landscape is being fundamentally reshaped by the recognition that nucleic acid circularity is not an anomaly but a core principle of molecular design employed by both host and pathogen. Endogenous circular RNAs have emerged from obscurity to be appreciated as a critical layer of gene regulation, intricately woven into the fabric of cellular homeostasis and innate immunity. Their engagement with the antiviral machinery is a study in contrasts, capable of both sounding the alarm against invaders and fine-tuning the intensity of the defensive response. This very system of host circRNA-based regulation provides a fertile ground for viral manipulation, with pathogens like EBV demonstrating a sophisticated ability to deploy their own circular RNAs to dismantle host immune surveillance.

Across the viral kingdom, a remarkable convergence toward circular nucleic acid architectures is evident. From the minimalist viroids to the complex episome of HBV, this topology confers undeniable advantages in stability, replication, and persistence. The Hepatitis B Virus cccDNA stands as the paramount example of this strategy, a formidable viral fortress in the hepatocyte nucleus that is responsible for lifelong infection and presents the single greatest obstacle to a cure. Our deepening understanding of the intricate network of host and viral factors that battle for control over cccDNA transcription is paving the way for targeted therapies and has driven the development of essential non-invasive biomarkers, like serum HBV RNA and HBcrAg, that allow us to monitor the activity of this hidden reservoir.

Finally, the translation of this fundamental knowledge into applied biotechnology is heralding a new era in medicine. The inherent stability and immunogenic properties of circular RNA are being harnessed to create a superior vaccine platform, promising more potent and durable protection against a host of viral diseases. In conclusion, the journey from viewing nucleic acids as exclusively linear molecules to appreciating the profound functional and clinical implications of their circular counterparts represents a significant paradigm shift. This expanded perspective continues to unravel new complexities in the ancient and ongoing arms race between viruses and their hosts and provides a powerful conceptual framework for designing the next generation of antiviral interventions.
